# Venom Profiling of a Population of the Theraphosid Spider *Phlogius crassipes* Reveals Continuous Ontogenetic Changes from Juveniles through Adulthood

**DOI:** 10.3390/toxins9040116

**Published:** 2017-03-25

**Authors:** Renan C. Santana, David Perez, James Dobson, Nadya Panagides, Robert J. Raven, Amanda Nouwens, Alun Jones, Glenn F. King, Bryan G. Fry

**Affiliations:** 1 Venom Evolution Lab, School of Biological Sciences, University of Queensland, St Lucia, QLD 4072, Australia; renan.castrosantana@uq.net.au (R.C.S.); davidcp@uci.edu (D.P.); james.dobson@uqconnect.edu.au (J.D.); nadya.panagides@gmail.com (N.P.); 2 Terrestrial Biodiversity, Queensland Museum, South Brisbane BC, QLD 4101, Australia; robert.raven@qm.qld.gov.au; 3 School of Chemistry and Molecular Biology, University of Queensland, St Lucia, QLD 4072, Australia; a.nouwens@uq.edu.au; 4 Institute for Molecular Bioscience, University of Queensland, St Lucia, QLD 4072, Australia; a.jones@imb.uq.edu.au (A.J.); glenn.king@imb.uq.edu.au (G.F.K.)

**Keywords:** tarantula, toxins, proteomic, mass spectrometry, LC/MS-MS, age

## Abstract

Theraphosid spiders (tarantulas) are venomous arthropods found in most tropical and subtropical regions of the world. Tarantula venoms are a complex cocktail of toxins with potential use as pharmacological tools, drugs and bioinsecticides. Although numerous toxins have been isolated from tarantula venoms, little research has been carried out on the venom of Australian tarantulas. We therefore investigated the venom profile of the Australian theraphosid spider *Phlogius crassipes* and examined whether there are ontogenetic changes in venom composition. Spiders were divided into four ontogenic groups according to cephalothorax length, then the venom composition of each group was examined using gel electrophoresis and mass spectrometry. We found that the venom of *P. crassipes* changes continuously during development and throughout adulthood. Our data highlight the need to investigate the venom of organisms over the course of their lives to uncover and understand the changing functions of venom and the full range of toxins expressed. This in turn should lead to a deeper understanding of the organism’s ecology and enhance the potential for biodiscovery.

## 1. Introduction

Spiders are abundant generalist predators that provide effective population control of other arthropods, most notably phytophagous insects [1]. They are found worldwide in all terrestrial ecosystems, except Antarctica. Araneae (Arthropoda: Arachnida) is one of the most speciose animal orders in the world [2], with >46,000 species, encompassing 4029 genera and 113 families [3]. Araneae is divided into two suborders, Mesothelae and Opisthothelae, with the latter suborder further subdivided into the infraorders Mygalomorphae and Araneomorphae. Mygalomorph spiders, known as primitive spiders, have two pairs of book lungs and a pair of parallel downward-facing chelicerae, each containing venom glands [4].

Theraphosidae is the most speciose mygalomorph family, with 140 genera encompassing 983 species [3]. Commonly known as tarantulas, bird-eating spiders, whistling or barking spiders, theraphosid spiders are the largest spiders in the world [5]. Like almost all spiders, they possess venom glands [6]. The main function of spider venom is prey acquisition, with predator deterrence as a secondary function [7,8,9]. Tarantula envenomations typically cause only mild symptoms in humans. However, species of the genera *Latrodectus, Loxosceles, Phoneutria, Atrax, Illawarra* and *Hadronyche* can cause sickness and even death [10,11].

Spider venom is a complex cocktail of different molecules including inorganic salts, small molecules such as amino acids, neurotransmitters and larger polyamines, peptides, and proteins [7,8,9]. The different compounds in spider venom are thought to work synergistically, thus increasing venom efficiency [9,12,13]. It has been estimated that each spider venom contains more than 100 peptides, with some containing more than 1000, which provides an immense natural pharmacopeia [10]. Spider venom composition may be influenced by different predatory niche factors such as sex, diet, habitat and climate, making the venom possibly species-specific and enlarging the opportunity for drug discovery [9]. Early studies of spider venoms focused on medically important spiders with a view towards development of antivenom [10]. Since the turn of the century, our increased understanding of the molecular composition of spider venoms and the mode of action of spider-venom compounds has led to the development of spider-venom peptides as pharmacological tools, drugs and bioinsecticides [14,15,16,17].

The venom ontogeny of spiders has been studied in species of the infraorder Araneomorphae, with a focus on differences related to size, sex and defense behaviour [18,19,20,21]. In contrast, although differences in venom composition between sexes have been investigated in mygalomorphs using modern molecular tools, ontogenetic variation in venom has only been studied using differences in LD_50_ and venom yield [22,23,24]. In other studies, focusing on different aspects of venom, ontogenetic variation in venom composition was only reported on briefly [25,26]. Thus, this study aims to provide, for the first time, a full venom proteomics characterization of ontogenetic variation within a population of mygalomorph spiders, namely the Australian tarantula *Phlogius crassipes* [27]. 

## 2. Results

### 2.1. LC-MS Analysis of Venom

LC-MS analysis of tarantula venoms was performed to provide a visual means of comparing the variation in venom composition between size groups. Peaks in the chromatogram correspond to the detection of a toxin or group of toxins with similar hydrophobicity. However, this method does not provide a comprehensive overview of venom complexity as most LC peaks will be comprised of multiple toxins.

**Figure 1:**
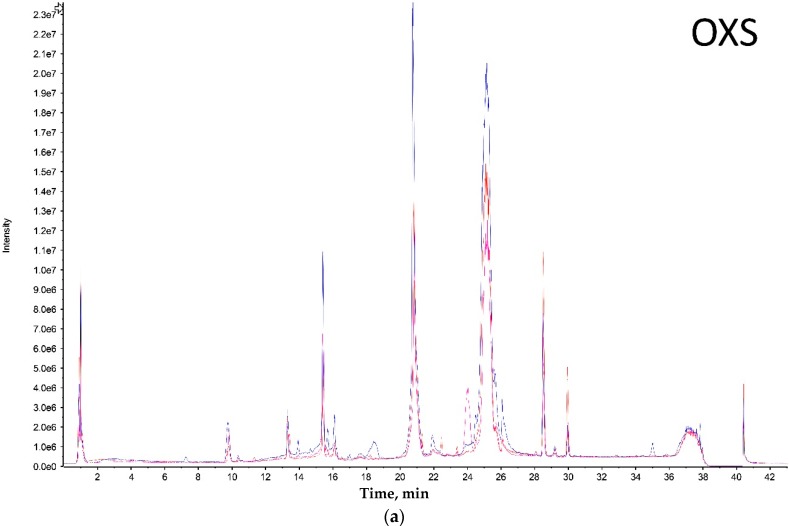

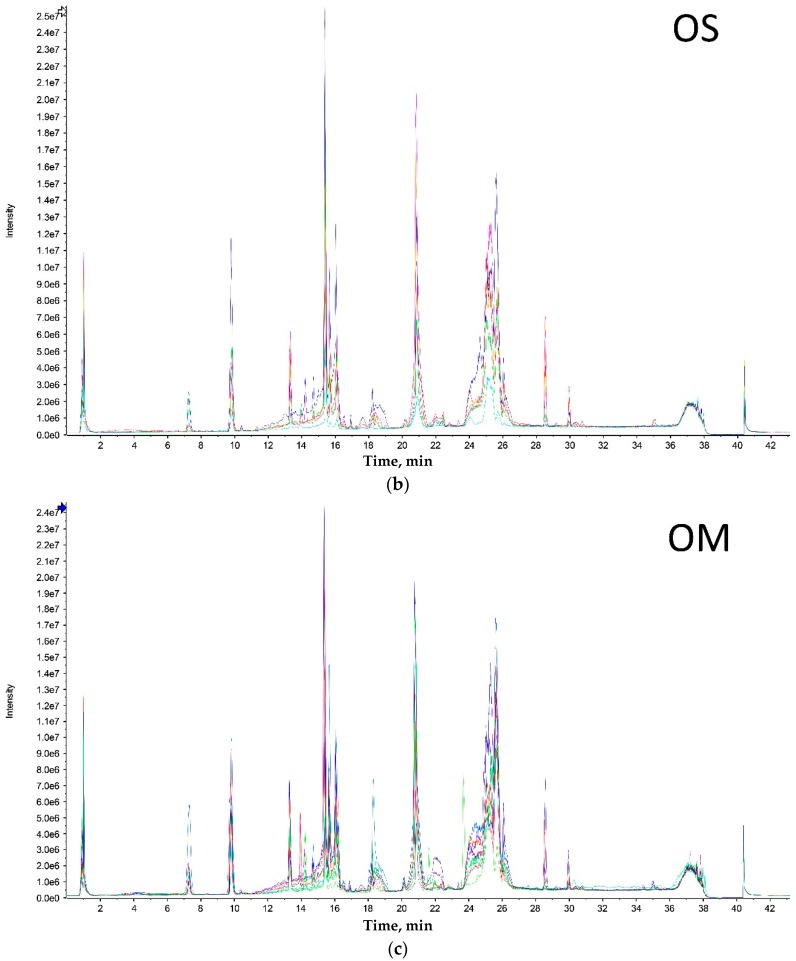

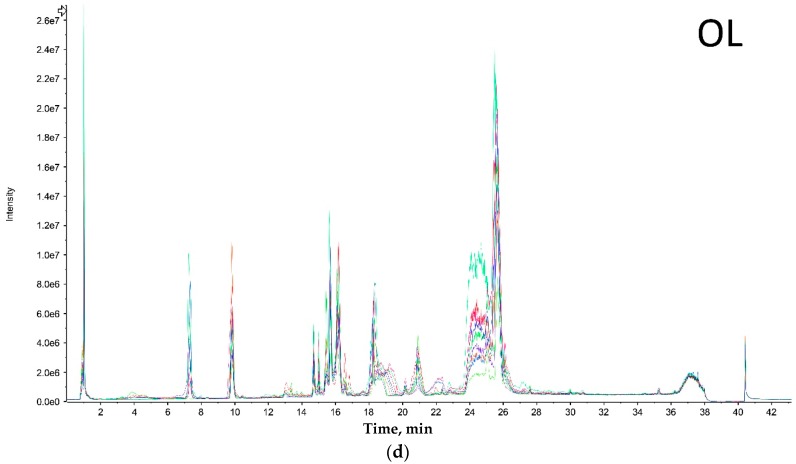
Combined liquid chromatography (LC) chromatograms of specimens from the (**a**) OXS – Olkola extra small group, (**b**) OS – Olkola small group, (**c**) OM – Olkola medium group, and (**d**) OL – Olkola large group. Each colour represent a different specimen. Vertical axis is Intensity and horizontal axis is Time (minutes).

Both extreme size groups showed a smaller number of peaks (Olkola extra small (OXS) and Olkola large (OL) with 9 peaks) (Figure 1a,d) compared with the intermediate groups (Olkola small (OS) and Olkola medium (OM) with 11 peaks) (Figure 1b,c). Small variations in intensity were noted in all chromatograms, but could be due to individual variation. Clear differences in peaks were observed between the smallest (Figure 1a) and largest (Figure 1d) groups while the intermediate groups (Figure 1b,c) did not differ significantly. Group OXS lacks the peaks around 7, 14, and 18 min that are observed in chromatograms from all other groups. Also, the OXS group has peaks of lower intensity at 10 and 15 min compared to its closest group (OS). Group OL lacks the peaks at 13, 28 and 30 min that are present in chromatograms from all other groups and it differs in the intensity of peaks at 15 and 21 min.

**Figure 2:**
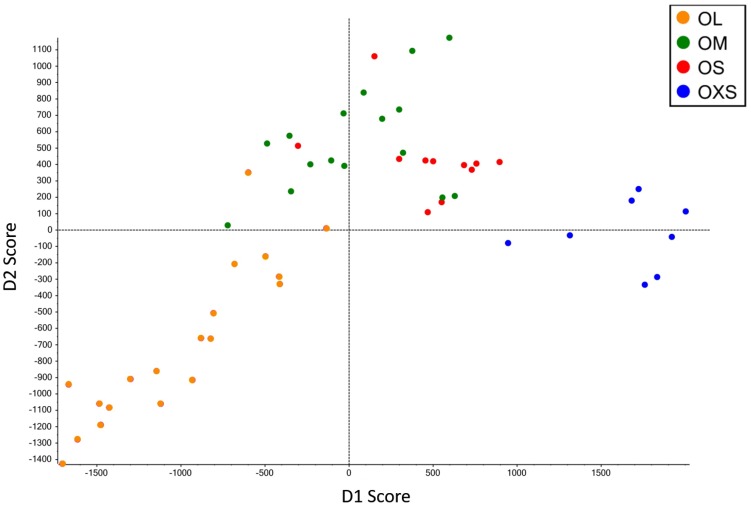
Principal component analysis and discriminant analysis (PCA-DA) analysis of *Phlogius crassipes* population from Olkola Aboriginal land. Tarantulas are grouped according to cephalothorax size. Each dot represents a specimen.

Principal component analysis and discriminant analysis (PCA-DA) (95.5%) showed a pattern of change in venom according to spider size (Figure 2), with gradual changes in venom pattern. However, it is clear that the venoms from tarantulas smaller than 10 mm (OXS) are different from tarantulas bigger than 10.1 mm (OS, OM and OL). Although specimens from the OS and OM groups are mixed in the central area of PCA-DA graph, OS spiders are more close to OXS spiders, while OM spiders are closer to OL. Adult tarantulas bigger than 15.5 mm (group OL) are dispersed over a larger area of the graph, but they overlap with OM in a small region.

### 2.2. Shotgun Sequencing of Venom Peptides and Proteins

LC-MS/MS of whole venoms (shotgun) was conducted to identify toxins found in each size group and determine if any changes in venom were occurring over the lifespan of *P. crassipes*. For a small number of spiders, it was difficult to extract a significant amount of venom. This was especially true for smaller specimens from the OXS and OS groups, which limited sample numbers for some analyses. The individuals selected to perform the shotgun analyses followed by LC-MS/MS were representative of each group.

MS analysis revealed a total of 12 peptides/proteins in the OXS group, 11 in OS, 8 in OM and 8 in OL. These results contrast with the LC/MS data presented above that showed more complexity in the venoms of OS and OM spiders (11 peaks), and less complexity in venom from OXS and OL spiders (9 peaks). However, as the LC/MS peaks can have one or more toxins with similar hydrophobicity overlapping in the chromatogram, the venom can be more complex in the early stages of life of tarantulas. On the other hand, the lack of a comprehensive database to match all the toxins in the venom may be responsible for the non-corroborative results between the shotgun and LC/MS analyses.

A BLAST of shotgun-derived sequences against the UniProt database revealed matches with almost 30 spider-venom components. Some of these were present in all of the *P. crassipes* samples, while others were found in just one representative tarantula. Almost 20 theraphosid toxins matched with the samples analysed (Table 1). Homologs of CRISP-2, U_3_-TRTX-Cg1a and μ-TRTX-Phlo1b toxins were found on all specimens. μ-TRTX-Phlo1b inhibits the human voltage-gated sodium channel Na_V_1.7, while the functions of U_3_-TRTX-Cg1a and CRISP-2 are still unknown. Clearly, the venom of small juveniles is more complex and includes a larger array of compounds, while large juveniles and female adults have less complex venom. However, some toxins are exclusive to each group, such as homologs of μ-TRTX-Phlo2a (OL), μ-TRTX-Phlo1a (OM), U_8_-TRTX-Hs1b (OS), U_1_-TRTX-Spl1a (OS), U_3_-TRTX-Cg1b (OS), U_1_-TRTX-Cv1a (OXS) and τ-TRTX-Gr1b (OXS).

All three μ-TRTX-Phlo toxins were described from another population of *Phlogius crassipes* [28], but we found only two of them in the larger individuals. U_1_-TRTX-Spl1a is found in venom from *Selenotypus plumipes*, a species that cohabits with a different population of *P. crassipes*. Jingzhaotoxin F7-15.33, two TRTX-Pg and the six TRTX-Cg toxins are found in the Asian tarantula *Chilobrachys guangxiensis*, while U_1_-TRTX-Cv1a is found in venom from the Asian tarantula *Coremiocnemis valida*, both from the same subfamily of the Australian tarantulas (*Selenocosmiinae*). The other three toxins were originally described from spiders in different subfamilies from Asia and America.

Shotgun similarities reveal that representatives of each spider group have different venoms, having a maximum of 40% similarity when compared to all peptides matched (Table 2). However, when only comparing against theraphotoxin matches, the similarity increased to 50%–80%. The venom of the spiders from small to large does not reflect a linear progression of juvenile venom to adult venom; rather, the venom changes without pattern between life stages. Venom of the group OL is more similar to the group OXS and OS than of the group OM on both tables. Permutation analysis also shows a statistical difference between all groups using the complete collection of peptides or just the matched theraphotoxins (Table 3).

Another 130 matches were found in the UniProt database (Supplementary Table S1). Most of these were peptides from the insect orders Diptera and Hymenoptera. Proteins from assassin bugs and ticks were also matched despite their more distant phylogenetic relationship to tarantulas and their different feeding methods. These matches were mostly to similarities with intracellular proteins or to similar toxins found in bees, wasps, ants, assassin bugs and ticks to the spider toxins. This could be an example of convergent evolution or recruitment of the toxin by the ancestors of these orders.

### 2.3. 1D SDS-PAGE Analysis of Venom

**Figure 3:**
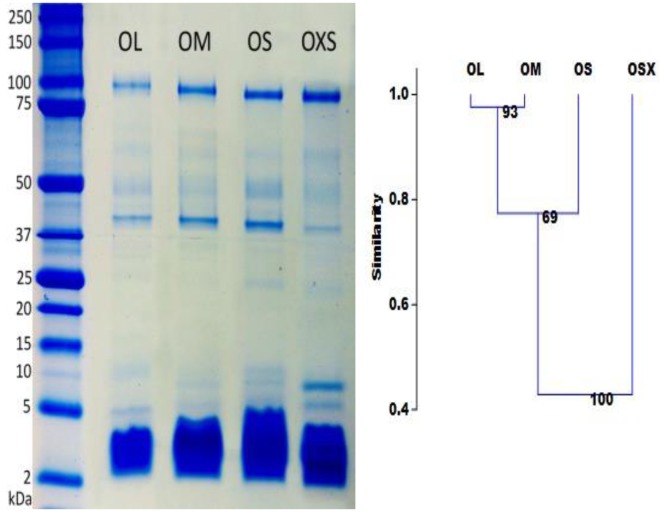
Left. 1D SDS PAGE gel of representatives from a population of *Phlogius crassipes*. Left lane is molecular marker (protein ladder) followed by OL, OM, OS and OXS specimens, respectively. Right. Clustering analyses with Rho similarity and 10,000 bootstrap values from one-dimensional gel electrophoresis of *P. crassipes* individuals of four different sizes.

While LC/MS separates toxins on the basis of hydrophobicity, 1D gel electrophoresis separates them according to molecular mass. The high molecular mass gel bands, which correspond to large proteins and enzymes, appear to be similar in all groups (Figure 3). However, the intensity of these bands differs between individuals, possibly indicating that these high molecular proteins are more important at different life stages than others.

We observed considerably more variation in the quantity and intensity of bands with molecular masses smaller than 25 kDa. The highest number of gel bands were found in the group OS (9 bands), followed by groups OXS and OM (8), and the group OL (7). These results are consistent with the shotgun analysis, which pointed to higher complexity for venom from the smaller spiders (groups OXS and OS). 

The intensity of the nine visible bands was examined using GelQuantNET software. Clustering results showed a gradual change in band intensity according to spider size (Figure 3). Group OXS appears to be the most different with just over 40% similarity with the other groups. The OS group is almost 40% different from OXS and 20% from OM and OL. OM and OL differs only 3%, but they are 50% different from OXS.

### 2.4. LC-MS/MS Analysis of 1D Gel Bands

In addition to the visual comparison that can be made from the ID SDS PAGE gels, LC-MS/MS was also conducted on the excised bands. The data provided by this analysis can detect lower abundance toxins missed in the shotgun analysis due to ion suppression. However, the shotgun analysis of venom can detect proteins and peptide often of lower molecular mass that cannot be resolved by the gel. The SDS PAGE gel and LC-MS/MS analysis of excised gel bands complement each other and provide a clearer picture of the venom that cannot be matched by the shotgun analysis.

**Figure 4:**
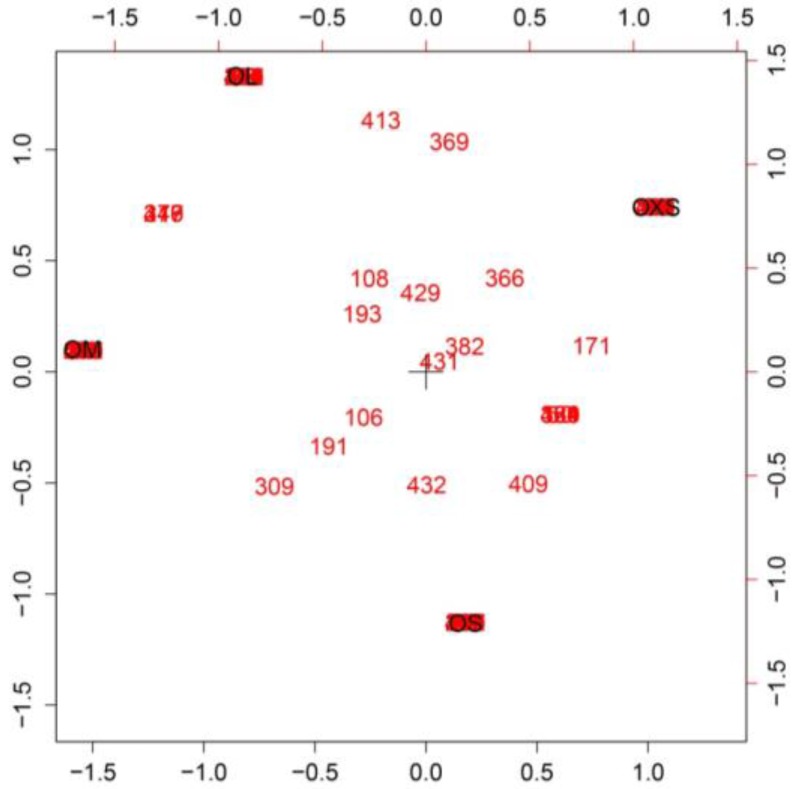
Simple Correspondence Analysis of representatives of group size from a population of *P. crassipes*. Each number corresponds to a single venom compound identified by Protein Pilot. Axes correspond to the two dimensions created by the analysis.

The Simple Correspondence Analyses showed a high number of peptides exclusive to each spider, with a few being found in all specimens (Figure 4). Some peptides were found in venom from two individuals as can be noted between OXS and OS, and between OM and OL.

Searches identified 443 protein matches in the UniProt database from all individuals and bands. Apart from a few matches to theraphotoxins, most of the proteins were from dipterans (flies, fruit flies and mosquitos), hymenopterans (ants, bees and wasps), assassin bugs and ticks. Interestingly, high molecular mass bands from OM and OXS spiders returned sequences that matched with Sictox-LhiαIA2a (*Loxosceles sp*.) and α-latrocrustotoxin (*Latrodectus sp*.), respectively. A match with CRISP-2 of *Grammostola rosea* was found in all specimens analysed except the representative from the OL group (Supplementary Table S2).

## 3. Discussion

A clear shift in venom profile is evident from the chromatograms obtained from the smallest (OXS) and largest (OL) spiders (Figure 1A,D). While visual inspection of the chromatograms appears to show that venoms of the OS and OM groups are most similar, cluster analysis revealed that OM venom is more closely related to that from OL spiders. Despite the lack of drastic variation in SDS PAGE gels of venoms from the four groups of spiders, the shotgun analysis revealed that the venom profile varies between each size group. In addition to venom changes from juvenile to adult, sampling of venom from older tarantulas showed that the venom continues to change even after adulthood, as evidenced in the PCA analysis (Figure 2).

Ontogenetic shifts in tarantula venoms have been reported previously [23,25,26]. Venoms of the Indian theraphosid spider *Poecilotheria rufilata* and the African theraphosid spider *Pterinochilus murinus* show variation between juveniles and adults, though this variation mostly resulted from changes in toxin abundance rather than differences in venom profile [25]. In addition, some ontogenetic changes recorded by this study may have been masked by the grouping of spiders according to sexual maturity, ignoring the size variation in juveniles to adults. Differences in both toxin abundance and venom profile were observed between juvenile and adult specimens in the Brazilian theraphosid spider *Lasiodora parahybana* [26]. However, the authors did not take into account spider size, but rather grouped spiders according to age, which may not reflect a change in prey size specialisation.

Spider size might, to some extent at least, determine the maximum-sized prey a tarantula can subjugate, which could lead to different micro-niches according to spider size. These different predator–prey interactions might impose different selection pressures that lead to variations in venom composition. Shifts in venom composition due to a change in diet have been observed in snakes where prey changes from one taxonomic class to another as the snakes mature [29,30,31]. The change in venom to target larger prey (i.e., small vertebrates in this case), might enable spiders to exploit more energy-rich resources as a trophic strategy.

The lack of sexually mature males in the sampling has made this research incomplete. Thus, changes in venom composition due to selection pressure exerted from exposure to different predators in adulthood was beyond the scope of this study but should be the subject of future work. Sexually mature male mygalomorph spiders are known to leave their burrows in search of females, exposing them to reptilian, avian and mammalian predators. Therefore, it would benefit the spider to have defensive toxins that target these animals in addition to their invertebrate-specific predatory compounds. For example, it was recently shown that venom from the tarantula *Heteroscodra maculata* contains algogenic peptides that induce pain in vertebrate predators and which are presumably used for defense [32].

The venoms of male tarantulas tend to show greater variation in composition compared to female venoms [22,23,25,33]. Herzig demonstrated that male *Coremiocnemis tropix* yield considerable less venom than females, but their venom contains a higher variety of toxin components [22,23]. However, in the South American tarantula *Acanthoscurria atrox*, female venom contains ~50% more compounds than male venom, with just 15% shared toxins between venom from both sexes [34]. *Brachypelma* and *Grammostola* venom differs quantitatively and qualitatively between sexes [25]. We predict that *P. crassipes* male venom likely has a different composition to the female venoms analysed here, as the ecology of sexually mature males differs from juveniles and adult females.

From a biodiscovery perspective, the lack of protein matches to the compounds identified by LC-MS/MS and shotgun analyses are due to the absence of comprehensive studies of tarantula venoms, especially Australian tarantulas. Transcriptome studies are needed to fully understand and characterize these spider venoms. With no transcriptome available for this species, identification of the venom proteome was limited. Moreover, without a transcriptome, false matches may occur. 

Researchers often use adult males or female spiders to find venom toxins that can be used to develop new drugs and bioinsecticides [6,35,36]. It is estimated that the venom of each species of spider contains at least 50 peptide toxins [37,38]. If the venom of a single species such as *P. crassipes* changes multiple times over its lifespan, the number of toxins with the potential for biodiscovery could be far greater. Therefore, studies are needed on the venom of juveniles to determine the full pharmacological potential of spider venoms. Researchers studying spider venoms should consider pooling venom from a spectrum of sizes/ages to obtain a better representation of the full range of toxins that can be expressed in the venom throughout the life of the animal.

## 4. Conclusions

This study revealed that *P. crassipes* venom changes continuously according to spider size, which we used as a proxy for age. Older Olkola spiders with a prosoma size over 15.5 mm continue to modify their venom composition even after becoming adults. Changes in venom composition during the lifetime of *P. crassipes* could be due to a change in the prey the spiders encounter at different life stages. Males are known to have an errant life stage where they leave their burrows in search of mates. During this time, the males are exposed to large mammalian and reptilian predators that they do not encounter when living in burrows or under logs and rocks. It is possible that their venom incorporates toxins that enable male spiders to defend themselves from predators during this life stage.

## 5. Materials and Methods

### 5.1. Spider Collection

*P. crassipes* specimens were collected at Olkola land, central Cape York, north Queensland. To ensure that all specimens collected were from the same population, samples were taken within a 1000 m radius from Killarney station (–15.425849, 143.495126). Thirty-four spiders of different sizes were sampled using active manual search during day and night. GPS coordinates were recorded when spider burrows were located. Spiders were collected under rocks and logs or excavated from burrows. This expedition was a component of the BushBlitz nature discovery program (bushblitz.org.au).

Spiders were transported alive to the Queensland Museum then stored in a dark room in containers containing moisturized sphagnum at a temperature of 24 ± 1 °C. They were fed house crickets (*Acheta domestica*) once per week. 

### 5.2. Venom Collection

Electrostimulation was used to extract the venom after one month of rehousing. Spiders were not fed for at least one week prior to venom extraction. The milking technique consisted of a forceps wire soldered to an adjustable electric converter that was used to stimulate the muscle surrounding the venom glands to contract and release venom. Voltage was 12 V for spiders with prosoma >13 mm, 9 V for spiders with prosoma of 10–13 mm, and 7.5 V for spiders with prosoma <10 mm. Venom was collected into a 1.5 mL microcentrifuge tube, lyophilized, then stored at –20 °C. 

### 5.3. Venom Proteomics

Spiders were separated into four groups according to cephalothorax length: group OXS ≤ 10 mm; 10 mm < OS > 13 mm; 13 mm ≤ OM ≥ 15.5 mm; and OL > 15.5 mm. These groups were defined by observation based on the size range of the food the spiders could safely handle. Lyophilized venoms were dissolved in 1 mL of MilliQ water and protein content quantified from absorbance at 280 nm measured on a NanoDrop 2000 spectrophotometer (Thermo Fisher Scientific, Waltham, MA, USA). Aliquots were then dried using a SpeedVac vacuum concentrator (Labconco, Kansas City, MO, USA) and stored at –80 °C until further processing.

#### 5.3.1. LC/MS (ESI uHPLC, Mass Spectrometry)

The extracts were analyzed by ESI LC-MS on a Shimadzu Prominence uHPLC (Kyoto, Japan; Canby, OR, USA) coupled to a Triple TOF 5600 mass spectrometer (SCIEX, Concord, ON, Canada) equipped with a duo electrospray ion source. 5 µL of each extract was injected onto a 2.1 mm × 100 mm Zorbax 300SB-C18 1.8 um column (Agilent, Santa Clara, CA, USA) at 300 µL/min. The samples were eluted from the HPLC column using a linear gradient of 2% solvent B for 1 min, 1%–40% solvent B over 35 min at 300 µL/min flow rate, followed by a steeper gradient from 40% to 98% solvent B in 5 min. Solvent B was held at 98% B for 2 min for washing the column and returned to 2% solvent B for equilibration prior to the next sample injection. Solvent A consisted of 0.1% formic acid (aq) and solvent B contained acetonitrile/0.1% formic acid (aq). The ionspray voltage was set to 5300 V, declustering potential (DP) 100 V, collision energy 8V, curtain gas flow 25, nebuliser gas 1 (GS1) 50, heater gas 2 (GS2) 50, gas temperature 500 °C and interface heater at 150 °C. The mass spectrometer acquired 500ms full scan Time-of-flight mass spectrometry (TOF-MS) data over the mass range 350–2000. The data was acquired and processed using Analyst TF 1.6 software (SCIEX, Concord, ON, Canada).

#### 5.3.2. In-Solution Sample Preparation for LC-MS/MS

For shotgun protein sequencing, whole crude venom from a representative individual of each group was reduced, alkylated, digested and submitted for LC-MS/MS analysis. This was performed by first dissolving 10 μg of lyophilized venom in 20 μL of 8 M urea. Samples were then reduced by incubation at 37 °C for 30 min following addition of 10 μL of 15 mM dithiothreitol (DTT) (Sigma-Aldrich, Sydney, Australia), then cysteine residues were alkylated by adding 10 μL of 100 mM iodoacetamide (IAA)(Bio-Rad) and incubating samples in the dark for 30 min at room temperature. 10 μL of 15 mM DTT was then added to quench excess IAA. 50 μL of 50 mM ammonium bicarbonate (ABC)(Sigma-Aldrich, Sydney, Australia) followed by trypsin (Sigma-Aldrich, Sydney, Australia) (1:50) were then added and the sample was incubated overnight at 37 °C to digest venom peptides and proteins. The samples were then desalted using a C18 ZipTip (EMD Millipore, Billerica, MA, USA) according to the manufacturer’s protocol. Dried samples were resuspended in mobile phase A (5% acetonitrile, 0.1% formic acid in deionised water).

#### 5.3.3. 1D SDS PAGE Non-Reduced and Reducing

1D gel electrophoresis was conducted using a representative individual from each group following a modified Laemmli protocol [39]; 40 μg of venom was loaded per lane, and gels were run at room temperature at 100 V until the loading dye reached approximately 10 mm from the base of the gel. “Non-reducing gels” were run using crude venom. “Reducing gels” were performed by dissolving venom samples in 5 μL of 3× sample loading buffer (15 μL total volume) with 50 mM DTT followed by 4 min incubation at 100 °C prior to loading onto the gel.

#### 5.3.4. GeLC-MS/MS

Samples were separated using RP-HPLC on a Dionex Ultimate 3000 RSLC nano-system (Lifetech, Carsbad, CA, USA). Samples were desalted on a Thermo PepMap 100 C18 trap (Lifetech, Carsbad, CA, USA) (0.3 × 5 mm, 5 µm) for 5 min using a flow rate of 30 µL/min, followed by separation on an Acclaim PepMap RSLC C18 (Lifetech, Carsbad, CA, USA) (150 mm × 75 µm) column at a flow rate of 300 nL/min. For bands cut out of 1D gels, a gradient of 10%–95% buffer B over 7 min was used, whereas for whole-venom samples processed using reduction/alkylation/ digestion (shotgun), a gradient of 10%–95% buffer B over 90 min was used. In both cases, buffer A was 1% ACN/0.1% FA and buffer B was 80% ACN/0.1% FA. Eluted peptides were directly analysed on an Orbitrap Elite mass spectrometer (Thermo Fisher Scientific, Waltham, MA, USA) using a nanospray ionization electrospray interface. Source parameters included a capillary temperature of 275 °C; S-Lens RF level at 60%; source voltage of 2 kV and maximum injection times of 200 ms for MS and 150 ms for MS2. Instrument parameters included an FTMS scan across the *m/z* range 350–1800 at 60,000 resolution followed by information-dependent acquisition of the top 10 peptides across the *m/z* range 40–1800. Dynamic ion exclusion was employed using a 15 s interval. Charge-state screening was enabled with rejection of +1 charged ions and monoisotopic precursor selection enabled. 

### 5.4. Bioinformatics and Statistical Analyses

LC-MS data were processed using the LC-MS peptides reconstruction tool in Analyst TF v1.6. LC graphs were edited to include the peak mass of the most relevant peaks. Combined LC graphs by groups were plotted using PeakView v2.2 (SCIEX). LC-MS files were input on Maker View v1.2.1 software (SCIEX), which generates an isotopes table for all samples. Isotope tables were classified according to elution time, intensity of peak and isotope mass. To avoid MS misreadings, isotopes that occurred in less than two samples were excluded and a maximum of 400 isotopes were selected to analyse. Marker View software performed a principal component analysis and discriminant analysis (PCA-DA).

Data from shotgun and 1D spot LC-MS/MS were converted to mascot generic format (mgf) using the msConvert software (ProteoWizard v3.0.9576) and Protein Pilot™ v5.0 (SCIEX) was used to search for sequence matches against all Arthropoda sequences (Uniprot and Trembl) obtained from www.uniprot.org downloaded on 21 January 2016. The Protein Pilot search conditions were set to include alkylation method (iodoacetamide), tryptic digestion, and allowing for urea denaturation and FDR analyses for shotgun and 1D gel modifications for 1D gel spots, including artefacts induced by the preparation or analysis processes. This was done to maximize the identification of protein sequences. Spectra were inspected manually to eliminate false positives. The list of toxins generated for each group were examined using Simple Correspondence Analyses [40,41].

Shotgun and 1D gel spots results were plotted on a separated binary matrix table, reflecting the presence and absence of peptides for each specimen. Peptides with more than 99% confidence for shotgun or 95% confidence for 1D spots and total coverage of minimum 2 for shotgun and 1 for 1D spots were included in the matrix. Similarity of Simpson were applied to verify percentage of difference between specimens using the freeware PAST v3.11 [42]. Shotgun matrix was analyzed by permutation of samples where performed and observed data were plotted against expected data using the Fossil package for R v3.2.3 [40,41]. 1D spots LC-MS/MS data matrix was analyzed by simple correspondence analyses using Mass package for software R v3.2.3 [43].

The intensities of 1D reduced gel spots were analyzed using the software GelQuantNET 1.8.2. Results were plotted as a matrix table showing the intensity of each spot from the gel. Clustering analyses using Rho similarity and 10,000 bootstrap samples were applied to verify the difference between specimens using the freeware PAST v3.11 [42].

## Figures and Tables

**Table 1:** Theraphotoxins from *P. crassipes* matched against UniProt arthropod database.

Mass	Toxins	OXS	OS	OM	OL
4,112	µ-theraphotoxin-Phlo1a				
4,155	µ-theraphotoxin-Cg1a				
4,146	µ-theraphotoxin-Phlo1b				
8,773	U_8_-theraphotoxin-Hs1b				
3,822	U1-TRTX-Spl1a				
8,869	U_36_-theraphotoxin-Cg1a				
3,284	µ-theraphotoxin-Phlo2a				
3,413	U_1_-theraphotoxin-Cv1a				
3,712	Jingzhaotoxin F7-15.33				
3,941	κ-theraphotoxin-Pg1b				
3,955	κ-theraphotoxin-Pg1a				
45,314	CRISP-2-*Grammostola rosea*				
47,520	Hyaluronidase (Fragment)				
6,940	κ-theraphotoxin-Cg3a 1				
3,681	δ-theraphotoxin-Cg1a 1				
4,334	U_3_-theraphotoxin-Cg1a				
4,366	U_3_-theraphotoxin-Cg1b				
4,150	τ-theraphotoxin-Gr1b				

**Table 2:** Left: Simpson’s similarity index for all peptides matched from the database. Right: Simpson’s similarity index for all theraphotoxins matched from the database (NA = not applicable).

	**OXS**	**OS**	**OM**	**OL**			**OXS**	**OS**	**OM**	**OL**
**OXS**	1.000	NA	NA	NA		**OXS**	1.000	NA	NA	NA
**OS**	0.400	1.000	NA	NA		**OS**	0.688	1.000	NA	NA
**OM**	0.300	0.300	1.000	NA		**OM**	0.700	0.700	1.000	NA
**OL**	0.300	0.300	0.233	1.000		**OL**	0.583	0.500	0.500	1.000

**Table 3:** Permutation analyses of shotgun binary matrix data. *p* > 0.05 indicates a significant difference. Left: permutation including all toxins matched by database search. Right: permutation including just spider toxins matched by database search (NA = not applicable).

	**OXS**	**OS**	**OM**	**OL**			**OXS**	**OS**	**OM**	**OL**
**OXS**	0	NA	NA	NA		**OXS**	0	NA	NA	NA
**OS**	0.9889	0	NA	NA		**OS**	0.3328	0	NA	NA
**OM**	0.9999	0.3918	0	NA		**OM**	0.3965	0.2194	0	NA
**OL**	0.9993	0.3901	0.5508	0		**OL**	0.7763	0.8015	0.3801	0

## Supplementary Materials

The following are available online at www.mdpi.com/2072-6651/9/4/116/s1, Supplementary Table S1: LC/MS-MS protein matches from reduced and digested venom, Supplementary Table S2: LC/MS-MS protein matches from 1D gel bands.

## References

[1] Roth M. (1993). Investigations on lead in the soil invertebrates of a forest ecosystem. Pedobiologia.

[2] Raizer J., Japyassú H.F., Indicatti R.P., Brescovit A.D. (2005). Comunidade de aranhas (Arachnida, Araneae) do pantanal norte (Mato Grosso, Brasil) e sua similaridade com a araneofauna amazônica. Biota Neotrop..

[3] World Spider Catalog. http://wsc.nmbe.ch.

[4] Framenau V.W., Baehr B.C., Zborowski P. (2014). A Guide to the Spiders of Australia.

[5] Coddington J.A., Levi H.W. (1991). Systematics and evolution of spiders (Araneae). Annu. Rev. Ecol. Syst..

[6] King G.F. (2004). The wonderful world of spiders: Preface to the special Toxicon issue on spider venoms. Toxicon.

[7] Escoubas P., Diochot S., Corzo G. (2000). Structure and pharmacology of spider venoms neurotoxins. Biochimie.

[8] Sannaningaiah D., Subbaiah G.K., Kempaiah K. (2014). Pharmacology of spider venom toxins. Toxin Rev..

[9] Vassilevski A.A., Kozlov S.A., Grishin E. (2009). V Molecular diversity of spider venom. Biochimie.

[10] Escoubas P., Sollod B., King G.F. (2006). Venom landscapes: Mining the complexity of spider venoms via a combined cDNA and mass spectrometric approach. Toxicon.

[11] Gray M.R. (2010). A revision of the Australian funnel-web spiders (Hexathelidae: Atracinae). Rec. Aust. Museum.

[12] Chan T.K., Geren C.R., Howell D.E., Odell G.V. (1975). Adenosine triphosphate in tarantula spider venoms and its synergistic effect with the venom toxin. Toxicon.

[13] Nagaraju S., Mahadeswaraswamy Y.H., Girish K.S., Kemparaju K. (2006). Venom from spiders of the genus Hippasa: Biochemical and pharmacological studies. Comp. Biochem. Physiol. Part C Toxicol. Pharmacol..

[14] King G.F. (2011). Venoms as a platform for human drugs: Translating toxins into therapeutics. Expert Opin. Biol. Ther..

[15] Smith J.J., Ho C., Lau Y.E. E., Herzig V., Ikonomopoulou M.P., Rash L.D., King G.F. (2015). Therapeutic applications of spider-venom peptides. Venoms to Drugs—Venom as a Source for the Development of Human Therapeutics.

[16] Saez N.J., Senff S., Jensen J.E., Er S.Y., Herzig V., Rash L.D., King G.F. (2010). Spider-venom peptides as therapeutics. Toxins.

[17] King G.F., Hardy M.C. (2013). Spider-venom peptides: Structure, pharmacology, and potential for control of insect pests. Annu. Rev. Entomol..

[18] Binford G.J. (2000). Diversification of Spider Venoms: Patterns and Correlates of Variation within Individuals and across Populations and Phylogenies. Ph.D. Thesis.

[19] Herzig V., Ward R.J., Dos Santos W.F. (2004). Ontogenetic changes in *Phoneutria nigriventer* (Araneae, Ctenidae) spider venom. Toxicon.

[20] De Andrade R.M.G., De Oliveira K.C., Giusti A.L., Da Silva W.D., Tambourgi D.V. (1999). Ontogenetic development of *Loxosceles intermedia* spider venom. Toxicon.

[21] Nelsen D.R. (2013). Ontogeny of Venom Use and Venom Composition in the Western Widow Spider *Latrodctus hesperus*. Ph.D. Thesis.

[22] Herzig V., Hodgson W.C. (2009). Intersexual variations in the pharmacological properties of *Coremiocnemis tropix* (Araneae, Theraphosidae) spider venom. Toxicon.

[23] Herzig V. (2010). Ontogenesis, gender, and molting influence the venom yield in the spider *Coremiocnemis tropix* (Araneae, Theraphosidae). J. Venom Res..

[24] Palagi A., Koh J.M.S., Leblanc M., Wilson D., Dutertre S., King G.F., Nicholson G.M., Escoubas P. (2013). Unravelling the complex venom landscapes of lethal Australian funnel-web spiders (Hexathelidae: Atracinae) using LC-MALDI-TOF mass spectrometry. J. Proteomics.

[25] Escoubas P., Corzo G., Whiteley B.J., Célérier M.-L., Nakajima T. (2002). Matrix-assisted laser desorption/ionization time-of-flight mass spectrometry and high-performance liquid chromatography study of quantitative and qualitative variation in tarantula spider venoms. Rapid Commun. Mass Spectrom..

[26] Guette C., Legros C., Tournois G., Goyffon M., Célérier M.L. (2006). Peptide profiling by matrix-assisted laser desorption/ionisation time-of-flight mass spectrometry of the *Lasiodora parahybana* tarantula venom gland. Toxicon.

[27] Koch L. (1984). Die Arachniden Australiens.

[28] Chow C.Y., Cristofori-Armstrong B., Undheim E.A.B., King G.F., Rash L.D. (2015). Three peptide modulators of the human voltage-gated sodium channel 1.7, an important analgesic target, from the venom of an Australian tarantula. Toxins.

[29] Andrade Denis V., Abe A.S. (1999). Relationship of venom ontogeny and diet in *Bothrops*. Herpetologica.

[30] Gibbs H.L., Sanz L., Chiucchi J.E., Farrell T.M., Calvete J.J. (2011). Proteomic analysis of ontogenetic and diet-related changes in venom composition of juvenile and adult Dusky Pigmy rattlesnakes (*Sistrurus miliarius barbouri*). J. Proteomics.

[31] Jackson T.N.W., Koludarov I., Ali S.A., Dobson J., Zdenek C.N., Dashevsky D., Op Den Brouw B., Masci P.P., Nouwens A., Josh P. (2016). Rapid radiations and the race to redundancy: An investigation of the evolution of Australian elapid snake venoms. Toxins.

[32] Osteen J.D., Herzig V., Gilchrist J., Emrick J.J., Zhang C., Wang X., Castro J., Garcia-Caraballo S., Grundy L., Rychkov G.Y. (2016). Selective spider toxins reveal a role for the NaV1.1 channel in mechanical pain. Nature.

[33] Rocha-e-Silva T.A., Sutti R., Hyslop S. (2009). Milking and partial characterization of venom from the Brazilian spider *Vitalius dubius* (Theraphosidae). Toxicon.

[34] Mourão C.B.F., Silva L.P., Block C., Schwartz E.F.N. (2007). Peptide profiling by matrix-assisted laser desorption-ionization time-of-flight mass spectrometry of the *Acanthoscurria atrox* tarantula venom: Evidences for sex-linked venom variation. IX Pan-American Section Congress of the IST.

[35] Herzig V., Hodgson W.C. (2008). Neurotoxic and insecticidal properties of venom from the Australian theraphosid spider *Selenotholus foelschei*. Neurotoxicology.

[36] Gentz M.C., Jones A., Clement H., King G.F. (2009). Comparison of the peptidome and insecticidal activity of venom from a taxonomically diverse group of theraphosid spiders. Toxicon.

[37] Escoubas P., Rash L. (2004). Tarantulas: Eight-legged pharmacists and combinatorial chemists. Toxicon.

[38] Tedford H.W., Sollod B.L., Maggio F., King G.F. (2004). Australian funnel-web spiders: Master insecticide chemists. Toxicon.

[39] Ali S.A., Yang D.C., Jackson T.N.W., Undheim E.A.B., Koludarov I., Wood K., Jones A., Hodgson W.C., McCarthy S., Ruder T. (2013). Venom proteomic characterization and relative antivenom neutralization of two medically important Pakistani elapid snakes (*Bungarus sindanus* and *Naja naja*). J. Proteomics.

[40] Vavrek M.J. (2011). Fossil: Palaeoecological and palaeogeographical analysis tools. Palaeontol. Electron..

[41] R Core Team (2016). R: A Language and Environment for Statistical Computing.

[42] Hammer Ø., Harper D.A.T., Ryan P. (2001). PAST: Paleontological Statistics Software Package for Education and Data Analysis. Palaeontol. Electron..

[43] Venables W.N., Ripley B.D. (2002). Modern Applied Statistics with S..

